# Correlation between Galectin-3 and Adverse Outcomes in Myocardial Infarction Patients: A Meta-Analysis

**DOI:** 10.1155/2020/7614327

**Published:** 2020-05-07

**Authors:** Lei Tian, Kan Chen, Zhihua Han

**Affiliations:** ^1^Department of Cardiology, Shanghai General Hospital, Shanghai Jiaotong University, 100 Haining Road, Shanghai, China; ^2^Department of Cardiology, Shanghai Ninth People's Hospital, School of Medicine, Shanghai Jiaotong University, 639 Zhizaoju Road, Shanghai, China

## Abstract

**Background:**

Acute myocardial infarction (AMI) is a disease with high morbidity and mortality. Some new biomarkers can help us to improve the life quality and prognosis of AMI patients.

**Objective:**

We therefore performed a systematic review and meta-analysis on the use of galectin-3 (gal3) for assessing prognosis of AMI patients.

**Methods:**

We searched Medline, Embase, Web of Science, Cochrane Library, SinoMed, China National Knowledge Infrastructure (CNKI), and Wanfang database up to June 2019. Trials included using galectin-3 to estimate prognosis in myocardial infarction (MI) patients.

**Results:**

We identified 10 trails with a total of 2809 participants. The negative correlation between galectin-3 and left ventricular ejection fraction (LVEF) was significant in 505 AMI patients (Fisher's *Z* −0.22, 95% CI: −0.34, −0.09). The correlation between galectin-3 and infarct size was not significant in 119 patients (Fisher's *Z* 0.12, 95% CI: −0.36, 0.60). Higher galectin-3 was associated with increased all-cause mortality in 2343 AMI patients (Fisher's *Z* 1.58, 95% CI: 1.23, 2.03).

**Conclusion:**

The limited evidence suggests that galectin-3 is likely to predict the adverse outcomes in MI patients, but it is not significantly correlated with infarct size after MI. More high-quality trials with longer-term follow-up are still needed to confirm this finding.

## 1. Background

Coronary artery disease (CAD) is one of the most common types of disease around the world. Although state-of-the-art treatment and multiple biomarkers can be used for early diagnosis and estimation of prognosis in CAD patients [1], some events of CAD such as acute myocardial infarction (AMI) are still associated with high morbidity and mortality [2].

Gal3 is a beta-galactoside binding lectin which can be produced by macrophages, vascular smooth muscle cells (VSMCs), and endothelium [3]. It has been found in the serum, cytoplasm, and nucleus and on the cell surface [3, 4]. Gal3 interacts with some receptors on the cell surfaces, and it can even be internalized directly by endocytosis in some kinds of cells [3]. Gal3 has been proved to be related to the fibrosis, atherosclerotic calcification, and cardiac remodeling [5, 6]. Gal3 is upregulated in the pathological process of atherogenesis [7]. Oxidized low-density lipoprotein (oxLDL) increases the expression of gal3 in VSMCs [4]. Gal3 also induces fibroblast and VSMCs to proliferate and produce fibrosis-related proteins in the extracellular matrix [8, 9]. Strategies to inhibit gal3 induce decreased atherosclerosis and may reduce plaque progression [7, 10].

The clinical use of gal3 for heart failure (HF) has been widely explored, and it is useful for the diagnosis and accurate estimation of prognosis in HF patients [11]. Although in some clinical studies, N-terminal prohormone of brain natriuretic peptide (NT-proBNP) might be superior to gal3 to diagnosis heart failure, gal3 is also widely considered as a novel biomarker to diagnosis heart failure [11–13]. The combination of gal3 with NT-proBNP was the best predictor for all-cause mortality and cardiovascular (CV) mortality in subjects with acute HF [14].

Myocardial infarction (MI) is one of the main causes that lead to HF, and some new biomarkers can help us to improve the diagnosis and prognosis of MI patients [15–17]. Recently, some clinical trials focus on the role of gal3 in MI patients and have demonstrated that gal3 is upregulated in these patients [18–20]. In AMI patients, gal3 boosts during the acute event and significantly decreases from baseline at the moment of discharge [17]. Gal3 is also suggested as a novel informative biomarker to predict adverse outcomes in MI patients [18, 21]. However, because there are some inconsistent results in these researches, we still do not certainly know the changes of gal3 and its roles in MI. The objective of this systemic review and meta-analysis is to determine the relationship between gal3 and adverse outcomes in AMI patients.

## 2. Methods

We included randomized controlled trials, prospective cohort studies, or case-controlled studies that enrolled MI patients. The eligibility studies had to report the following results: (1) gal3 levels and clinical outcomes during follow-up and (2) the Pearson or Spearman correlation coefficient between gal3 and cardiac function (LVEF or infarct size). Echocardiography or cardiac magnetic resonance (CMR) imaging functional data were extracted; echocardiography data were preferentially used unless MRI data were available. The serum gal3 was measured with the ELISA; therefore, we excluded studies where the participants were chosen from community or gal3 was measured by using some other methods instead of ELISA.

The following electronic databases were searched for the original review: PubMed, Web of Science, SinoMed, China National Knowledge Infrastructure (CNKI), and Wanfang database. Keywords included “galectin3,” “cardiovascular disease,” “coronary heart disease,” “acute coronary system,” and “myocardial infarction.” Medical subject headings (MeSH) or equivalent and text word terms were used. The publications written in English or Chinese were included in our search.

Two review authors independently reviewed titles and abstracts of all studies identified by the electronic searches and retrieved potentially relevant studies. We then read the full text of relevant studies and excluded any study that did not satisfy the inclusion criteria. All disagreements were resolved by discussion with a third review author until a consensus was achieved. In case of missing or unclear data for our analysis, we contacted the corresponding authors to clarify or get the data that were missing.

In order to get the standard error (SE) depending on the value of the Pearson correlation coefficient, a Fisher transformation was used to convert each correlation coefficient into an approximately normal distribution. The inverse variance formula was used to calculate the data [22]. For each outcome, tests of heterogeneity were carried out by using RevMan 5.3 (the chi-squared test of heterogeneity and the I^2^ statistic). We used the random effects model (Mantel-Haenszel (M-H) method) to pool the data.

Two authors independently assessed the risk of bias of included studies according to the Cochrane Handbook (Version 5.1.0) and rated each domain as having a low risk of bias, a high risk of bias, or an unclear risk of bias. Disagreements were settled by discussion or consulted a third review author.

## 3. Results

**Figure 1:**
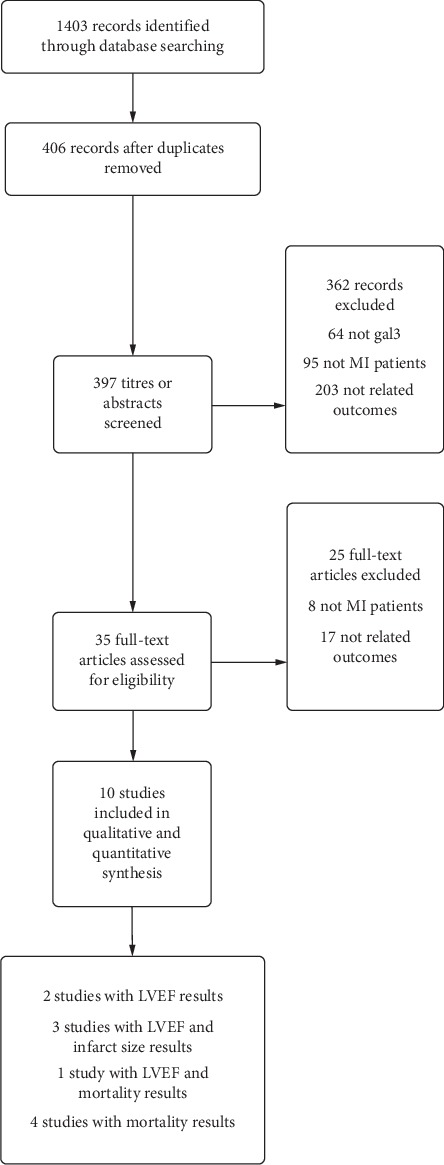
Flowchart showing the number of papers identified, screened, and included in the meta-analysis.

The database searches generated 419 (PubMed), 826 (Web of Science), and 158 (SinoMed, CNKI, and Wanfang database) hits and 406 after reduplication. Screening the titles and abstracts identified 35 papers for formal inclusion or exclusion, of which 10 papers met the inclusion criteria. Details of the screening process through the review are given in Figure 1. Key characteristics of included articles are described in Table 1.

Eight studies recruited participants with MI (Mayr 2012 [23]; Szadkowska 2013 [24]; Tsai 2012 [2]; Weir 2013 [25]; Singsaas 2016 [26]; Lisowska 2016 [18]; Di Tano 2017 [27]; Gagno 2019 [28]; and Asleh 2019 [29]), and the remaining one study included MI and chronic stable angina/microvascular angina patients (George 2015 [30]). Because in Singsaas's study, data were separately reported in two different patient groups (group1: at 1 years after MI; group2: at 4.4 years after MI), we treated the data from two different patient groups in our analysis as two studies. The duration of the follow-up periods varied between 3 days and 4.4 years.

### 3.1. Left Ventricular Ejection Fraction (LVEF)

**Figure 2:**
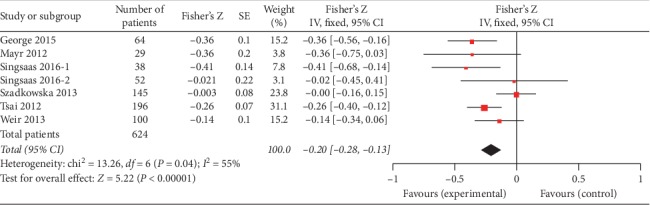
Forest plot between gal3 and LVEF using a random effects model.

Seven studies provided the *r* value between gal3 and LVEF (624 patients). The pooled results of these studies are shown in Figure 2. It revealed that gal3 was statistically correlated to the LVEF (Fisher's *Z* −0.22, 95% CI: −0.34, −0.09) with a relatively high level of heterogeneity (I^2^ = 55%, *P*=0.04). We conducted subgroup analysis to explore the statistical heterogeneity.

**Figure 3:**
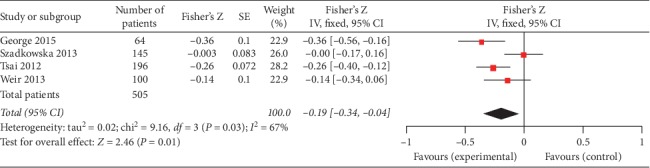
Forest plot of between gal3 and LVEF during AMI using a random effects model.

Four studies (505 patients) provided the *r* value between gal3 and LVEF which were measured during AMI. The pooled results of these four studies revealed that the negative correlation between gal3 and LVEF was statistically significant (Fisher's *Z* −0.19, 95% CI: −0.34, −0.04), but there was a high level of heterogeneity (I^2^ = 67%, *P*=0.03). The forest plot between gal3 and LVEF is shown in Figure 3.

**Figure 4:**
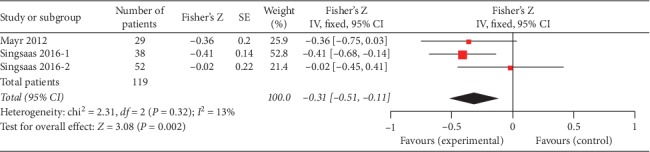
Forest plot of between gal3 and LVEF at long time after MI using a random effects model.

Three studies (119 patients) reported the data of gal3 and LVEF which were measured at relatively long time after MI (Mayr 2012 4 months and Singsaas 2016 1 year and 4.4 years). There was a statistically significant negative correlation between gal3 and LVEF (Fisher's *Z* −0.31, 95% CI: −0.52, −0.09).  Figure 4 shows the forest plot.

### 3.2. Infarct Size

**Figure 5:**
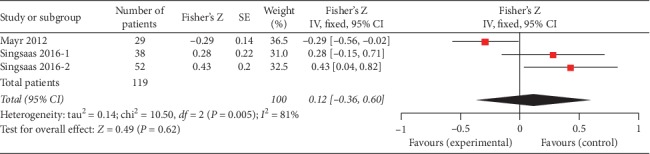
Forest plot of between gal3 and infarct size using a random effects model.

We also found that three studies (119 patients) evaluated the association between gal3 and infarct size (Mayr 2012, 4 months; Singsaas 2016, 1 year and 4.4 years). Although these three studies showed negative correlation between gal3 and infarct size, the overall effect was not significant (Fisher's *Z* 0.12, 95% CI: −0.36, 0.60). The forest plot is shown in Figure 5.

### 3.3. Mortality or HF Onset

**Figure 6:**
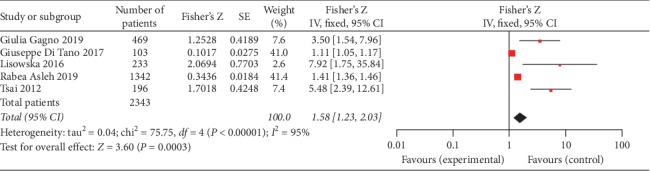
Forest plot of between gal3 and all-cause mortality using a random effects model.

MI is one of the common diseases with high morbidity and mortality. It was our interest to explore the effectiveness of gal3 to predict the mortality or HF onset after MI. We found that five studies (2343 patients) evaluated the association between gal3 and mortality or HF onset (Tsai 2012; Lisowska 2016; Gluseppe Di Tano 2017; Gagno 2019; and Asleh 2019). Our pooled results indicated that higher gal3 was associated with increased all-cause mortality in MI patients (Fisher's *Z* 1.58, 95% CI: 1.23, 2.03), and there was a high level of heterogeneity (I^2^ = 95%, *P* < 0.01). The forest plot is shown in Figure 6.

## 4. Discussion

CAD is one of the most common types of disease around the world. Single biomarkers as well as different combinations of biomarkers have been proven to be of some utility in defining prognosis in MI patients [31–33]. Data on the usefulness of the integrated use of gal3 in the diagnosis and prognosis of MI are lacking. Some recent studies have explored the role of gal3 in MI patients; hence, to comprehensively assess the diagnosis and prognosis role of gal3 in MI patients, we conducted this exhaustive meta-analysis. In this article, we elevated to use meta-analysis to pool different studies together and summarized our viewpoints in this field.

LVEF, frequently used for assessment of left ventricular, is an important parameter for predicting an unfavorable clinical outcome in AMI patients. After acute myocardial injury, LV remodeling is more or less happened, and a series of mechanical and neurohormonal factors may take part in this process that results in the progressive deteriorate of LVEF [21], leading ultimately to the major adverse cardiovascular events (MACE). In our included articles, we found that four studies examined the LVEF during the acute period of MI, and three studies measured the LVEF at a relatively long time after MI, so we analysed them separately. Although the correlation is a little weak, our pooled results indicated that gal3 had a significantly negative correlation with LVEF (*r* = −0.20, 95% CI: −0.27,−0.13).

The meta-analysis showed that gal3 significantly predicted the decline of LVEF, but there was a high level of heterogeneity (I^2^ = 53%, *P*=0.05) in this analysis. One of the main reasons was that the time of detecting LVEF and collecting the serum sample were not permanent. In Szadkowska's study, peripheral blood samples were collected within 36–60 h after acute MI, and LVEF was assessed on the 3^rd^–5^th^ day of MI [24]. In Weir's study, the mean time from AMI to performing gal3 was 46 hours and to screening transthoracic echocardiographic was 34 hours [25]. George reported the blood samples were collected within 48 hours of admission to the cardiac intensive care unit (CICU) [30], while Tsai collected the blood samples within 18 hours after AMI and measured the LVEF on day 2 following AMI [2]. Blood samples were obtained four months after the acute event in Mayr's study [23]. Singsaas tried to explore the relationship between gal3 and LVEF at 1 year and 4.4 years after MI [26]. Here, subgroups were distinguished based on the timing between the onset of AMI symptoms and the first measurement. We found that heterogeneity was high in the subgroup of AMI, but low in the subgroup of relatively long time after MI.

Infarct size is a major parameter of cardiac function after MI [26, 34] and, together with LVEF, is an important predictor of MACE [26]. Here, we found 3 studies with 177 participants meet our inclusion criteria. Our analysis indicated that serum gal3 was not significantly negatively correlated with infarct size in MI patients according to the *P* value (*P*=0.87). However, the results should be interpreted seriously because we just included 3 studies in this analysis. More importantly, the time of collecting the blood sample and performing cardiac magnetic resonance (CMR) imaging was different.

MI, one subtype of CAD, is one of the major causes of HF characterized by high morbidity and mortality. It was our interest to explore the value of gal3 for predicting MACE and mortality in these patients. We found that three studies examined the association between gal3 level and death [18, 28, 29]. We also found two clinical trials which reported similar outcomes in MI patients, one reported the value of gal3 in prediction of 30 day major adverse clinical outcome (MACO) [2], and the other analysed the value of gal3 for predicting all-cause mortality [18]. The value of gal3 significantly predicted the primary outcome of these clinical trials, and it seems that increasing gal3 is related to the adverse cardiovascular diseases. Although the correlation is little weak, our pooled results indicated that gal3 had a significant correlation with all-cause mortality in MI patients (Figure 6).

Although we endeavored to search out all relevant studies, until now there are only a small number of trials included in this review; besides, the most of the studies in our review included relatively limited numbers of participants. Particularly, there was a variation in the status of participants between studies. Most of the studies included a varied range of follow-up time that might have a bearing on these results. In the future, we still need more studies with long follow-up time to further assess the value of gal3 in the aspect of prognosis of MI.

The limited trial evidence suggests gal3 is statistically negatively correlated with LVEF in MI patients. More importantly, the available trials are supportive of favourable effects of gal3 on predicting mortality or LVEF after MI. However, in this field, there is still one large on-going trial in which gal3 concentration increased risk of all-cause mortality in MI patients during mid-term follow-up [18]. High-quality trials with long-term follow-up are necessary to determine the value of gal3 on predicting cardiac function in MI patients.

## 5. Conclusion

Serum gal3 has been regarded as a novel biomarker to predict long-term adverse outcomes in HF patients; however, the role of gal3 in diagnosis and assessing the prognosis of MI is still controversial. In our study, we found that gal3 was statistically correlated to the LVEF with a relatively high level of heterogeneity. In AMI patients, the negative correlation between gal3 and LVEF was statistically significant, but there was a high level of heterogeneity. After a relatively long time of MI, there was the statistically significant negative correlation between gal3 and LVEF, and there was a low level of heterogeneity. The negative correlation between gal3 and infarct size was not significant. There was a high level of heterogeneity. More importantly, our pooled results indicated that higher gal3 is related to the increased all-cause mortality in MI patients during the follow-up period between 30 days and 5.4 years. The limited evidence suggests that gal3 is likely to predict the adverse outcomes and LVEF in MI patients, but it is not significantly correlated with infarct size after MI. We are still looking forward to more and more reliable and persuasive clinical trials in the future.

## Figures and Tables

**Table 1:** Summary details of included studies.

Study	Publication year	Mean follow-up	Number of participants	Type of participants	Observational results
Asleh	2019	5.4 years	1342	Myocardial infarction	Mortality
Di Tano	2017	18 months	103	Myocardial infarction	Mortality
Gagno	2019	12 months	469	Myocardial infarction	Mortality
George	2015	6 months	102	Chronic stable angina, microvascular angina, and myocardial infarction	LVEF
Lisowska	2016	2.8 years	233	Myocardial infarction	Mortality
Mayr	2012	4 months	29	Myocardial infarction	LVEF and infarct size
Singsaas1	2016	4.4 years	52	Myocardial infarction	LVEF and infarct size
Singsaas2	2016	1 year	38	Myocardial infarction	LVEF and infarct size
Szadkowska	2013	3–5 days	145	Myocardial infarction	LVEF
Tsai	2012	30 days	196	Myocardial infarction	LVEF and mortality
Weir	2013	24 weeks	100	Myocardial infarction	LVEF
